# Interstitial Fibroid of Uterus

**Published:** 1895-07

**Authors:** Edmund Carleton

**Affiliations:** 53 West 45th St., New York, N. Y.


### INTERSTITIAL FIBROID OF UTERUS.

53 WEST 45TH ST., NEW YORK, N. Y.
MAY 15th, 1895.

TO THE EDITOR OF THE HOMŒOPATHIC PHYSICIAN:

In the published volume of *Transactions of the International Hahnemannian Association*, 1894, page 188, middle paragraph, occurs the following:

"Again, we have hereditary conditions in which we cannot trace the cause to any special miasm. Here with Tuberculinum in a very high potency, based upon the symptoms given by Burnett, I not only stopped the growth of a large interstitial fibroid of the uterus, but it is slowly disappearing. This case has been examined by some of the best surgeons of our school, among them Dr. Carleton, of New York, and considered incurable. Two years ago the patient was confined to her bed a good deal of the time. Now she looks after all her household cares unassisted. The growth followed the suppression of a leucorrhœa by medicated injections, the nature of which could not be ascertained."

Attention is called to the following correction of the above: I recollect the case, and my opinion of it given at the time of examination. Instead of considering it incurable I advised that the patient should be kept constantly under homœopathic treatment until the menopause should have been passed (the woman was already middle-aged), when absorption and recovery would probably follow.

Feeling sure that all concerned will be glad to have a slip of the pen set right, I am, yours truly,

EDMUND CARLETON.

